# Edward Sacher (1934–2023): Understanding Chemistry at the Interface

**DOI:** 10.3390/molecules31173088

**Published:** 2026-09-03

**Authors:** Michael Sacher

**Affiliations:** Department of Biology, Concordia University, Montreal, QC H4B1R6, Canada; michael.sacher@concordia.ca

**Keywords:** surface chemistry, interface chemistry, X-ray photoelectron spectroscopy, polymer metallization, nanomaterials, biomaterials

## Abstract

Edward Sacher (1934–2023) devoted more than five decades to understanding the chemistry of surfaces and interfaces. Trained as a physical chemist, he applied fundamental chemical principles to increasingly diverse materials, from polymers and metal–polymer interfaces to nanoparticles, carbon materials, and biological systems. A central theme of his work was the use of surface analytical methods, particularly X-ray photoelectron spectroscopy (XPS), not simply to characterize materials but to reveal the chemical interactions governing their properties and performance. His research connected fundamental interfacial physical chemistry with applications in materials science, microelectronics, nanotechnology, and biomedicine. This commentary traces the development of that scientific program, highlighting selected contributions as well as the laboratory, collaborations, and scientific community he built at École Polytechnique de Montréal.

## 1. Introduction

Few questions in materials science are as fundamental as what occurs when two materials come into contact. Whether the interface is between a metal and a polymer, a catalyst and its support, or a biomaterial and living tissue, the chemistry occurring within only a few atomic layers frequently determines the performance of the entire system. Throughout a scientific career spanning more than five decades, Edward Sacher sought to understand those interfacial interactions. Although the materials he studied evolved with the field—from polymer films and thin metallic coatings to carbon nanotubes, nanoparticles, graphene, and biomaterials—the central scientific question remained remarkably consistent: how does chemistry at a surface or interface govern material behaviour?

Edward Sacher’s career coincided with a period during which surface science evolved from a specialized discipline into a cornerstone of modern materials research. Improvements in vacuum technology, electron spectroscopy, and surface analytical methods made it possible to investigate interfaces with an unprecedented level of chemical detail. Edward recognized early that these techniques could provide far more than elemental analysis. Used thoughtfully, they could reveal the chemical interactions responsible for adhesion, reactivity, electronic structure, and ultimately material performance. Throughout his career he consistently applied fundamental chemical principles to technologically important problems, helping establish surface analysis—and particularly X-ray photoelectron spectroscopy (XPS)—as an indispensable tool for understanding materials.

His scientific contributions extended well beyond his own publications. He established one of Canada’s leading academic laboratories devoted to surface and interface chemistry, organized international scientific meetings, fostered collaborations across North America, Europe, and Israel, and trained graduate students and postdoctoral fellows who carried his approach into academia and industry. More importantly, he demonstrated that careful chemical analysis of surfaces could answer questions that were central to polymer science, electronics, nanotechnology, and later biomaterials. This article traces the evolution of that scientific program and the enduring influence it has had on the field. The major stages in the evolution of Edward’s scientific program, together with selected contributions and representative publications, are summarized in Table 1 and discussed in greater detail below.

## 2. A Chemist’s Foundation

Edward Sacher was born in New York City in 1934. Following graduation from Stuyvesant High School in 1951, he attended The City College of the City of New York, receiving a Bachelor of Science degree in Chemistry in 1956. He then entered graduate studies at The Pennsylvania State University, where he specialized in physical chemistry while completing a minor in organic chemistry. His doctoral dissertation, *Studies in the Theory of Acidity Functions*, completed in 1960 under the supervision of Norman C. Deno, addressed the quantitative description of acidity in strongly acidic media [1].

Although the subject matter appears far removed from the surface chemistry that later defined his career, the dissertation already reveals many of the characteristics that distinguished his scientific approach. Rather than treating experimental observations descriptively, Edward sought quantitative explanations rooted in fundamental chemical principles. The emphasis on molecular interactions, equilibrium, and careful interpretation of experimental evidence would remain evident throughout his later work, even as his research shifted toward increasingly complex materials systems.

His scientific development was further shaped by two distinguished postdoctoral appointments. At The Ohio State University he worked with Melvin S. Newman, whose pioneering studies of molecular conformation transformed modern organic chemistry. He subsequently joined Keith J. Laidler at the University of Ottawa, one of the founders of modern chemical kinetics. These mentors represented three complementary traditions within chemistry: mechanistic physical organic chemistry, molecular structure, and quantitative kinetics. Together they provided Edward with an unusually broad intellectual foundation that would later allow him to move comfortably between chemistry, physics, engineering, and materials science.

Looking back, it is striking how little his scientific philosophy changed over the decades. The systems became increasingly sophisticated, but the questions remained rooted in chemistry. This perspective distinguished him from many contemporaries who approached materials primarily from an engineering perspective. Edward approached materials as a chemist, convinced that macroscopic properties could only be understood by first understanding the molecular interactions that produced them.

## 3. Industry and the Emergence of a Scientific Program

Following his postdoctoral training, Edward entered industrial research, first with DuPont and subsequently at the IBM Materials Laboratory, where he worked from 1968 until 1981. His research at IBM encompassed the analysis of polymeric materials and interfacial phenomena. Among his publications from this period was a series examining water transport through polymer films, including polyimides and fluoropolymers. These studies considered diffusion and adsorption processes and reflected an interest in the relationship between the chemical and physical properties of polymers and their behaviour [2].

When Edward joined École Polytechnique de Montréal in the early 1980s, surfaces and interfaces became an increasingly prominent focus of his research. Early work examined bonding and adhesion at metal–polymer interfaces, an area that developed into a substantial program in polymer metallization. By the end of the decade, he had organized an international American Chemical Society symposium on the subject, followed by the volume *Metallization of Polymers*, which he edited with Jean-Jacques Pireaux and Steven P. Kowalczyk of IBM. His own contribution to that volume examined the chemistry underlying adhesion between deposited metals and polyimide surfaces. This work also connected fundamental studies of interfacial physical chemistry with the practical requirements of microelectronics, where the control of metal–polymer interfaces was becoming increasingly important to integrated-circuit metallization. Polymer–metal interfaces thus became an important component of the broader program in surface chemistry that he would pursue over the following decades [3].

## 4. Building a Centre for Surface Science

One of Edward Sacher’s most enduring contributions was the creation of an internationally recognized research program in surface and interface chemistry at École Polytechnique de Montréal. At a time when advanced surface analytical instrumentation remained relatively uncommon in Canadian universities, he recognized that meaningful advances in materials chemistry would increasingly depend upon the ability to characterize surfaces with atomic-scale chemical precision.

In 1984 he founded the Regional Surface Laboratory, serving as its Director for many years before later becoming Associate Director. The laboratory brought together an exceptional suite of surface analytical techniques, including X-ray photoelectron spectroscopy (XPS), ultraviolet photoelectron spectroscopy (UPS), Auger electron spectroscopy, time-of-flight secondary ion mass spectrometry (TOF-SIMS), and photoacoustic Fourier-transform infrared spectroscopy. These capabilities allowed researchers to investigate interfacial chemistry using complementary techniques rather than relying upon a single analytical method. 

The laboratory quickly became a focal point for collaborative research. Students, visiting scientists, and collaborators were drawn not only by the instrumentation itself, but by the scientific expertise required to interpret the results. As Edward later summarized in an NSERC research statement, the laboratory developed into one of the leading academic centres devoted to interfacial reactions, attracting researchers from around the world and supporting long-standing collaborations with laboratories in Belgium, Israel, the United States, and across Canada. 

Edward also helped build an international community around surface chemistry. The American Chemical Society invited him to organize international symposia on interfacial reactions in Montréal in both 1989 and 2001, meetings that brought together many of the leading researchers in the field and resulted in edited proceedings volumes that documented advances across a rapidly evolving discipline. 

**Figure 1:**
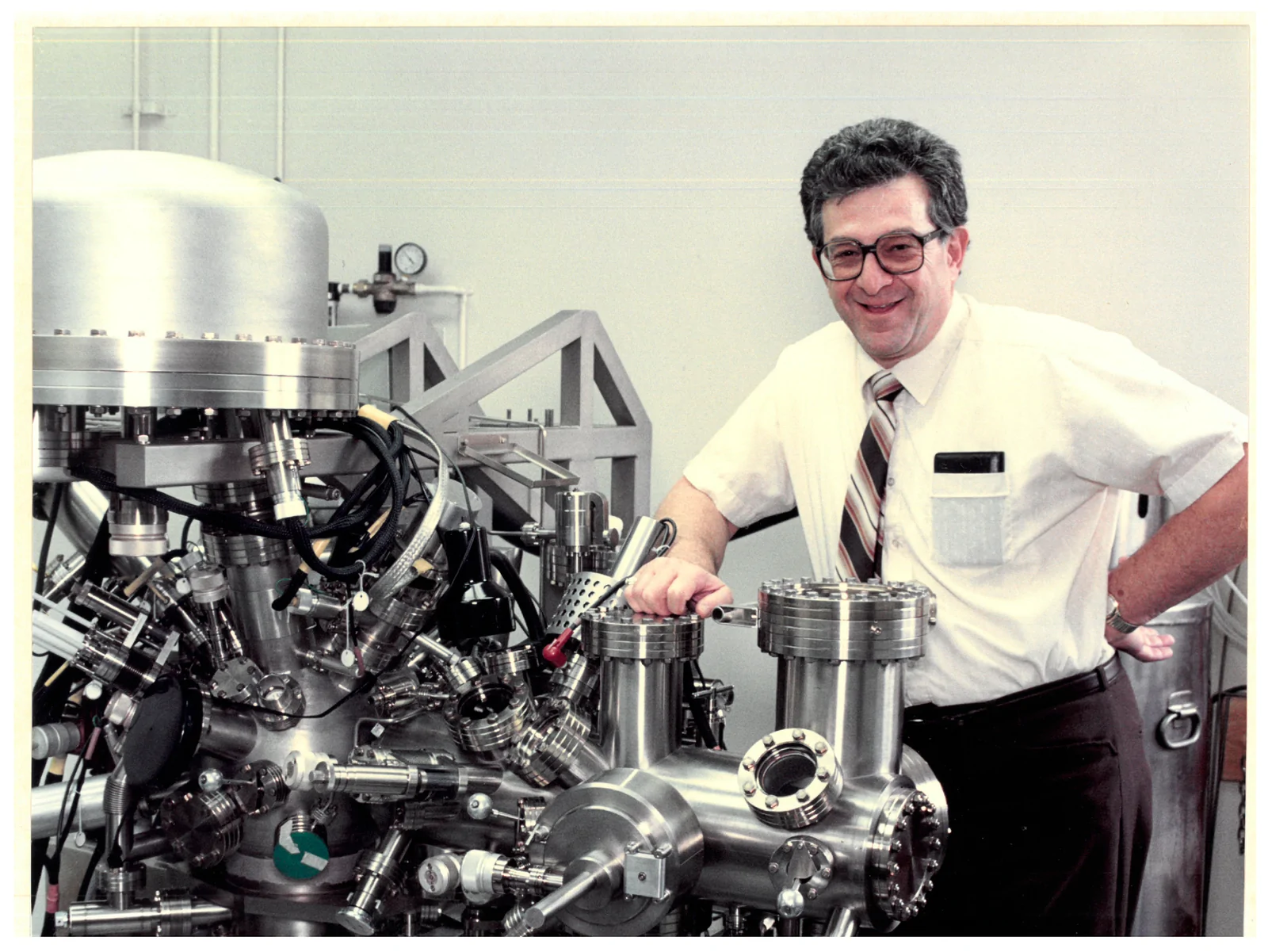
Edward Sacher (circa 1985) with the X-ray photoelectron spectroscopy (XPS) system in the Regional Surface Laboratory at École Polytechnique de Montréal. Photograph from the Sacher family collection; reproduced with permission of the Sacher family.

Figure 1 captures this period of his career. The photograph shows Edward standing beside the XPS instrument that became the centrepiece of the Regional Surface Laboratory. The instrument represented far more than a major acquisition for the laboratory; it provided the capability to address scientific questions that previously could not be approached experimentally. Much of the research that followed, from polymer interfaces to nanomaterials and biomaterials, was made possible by the capabilities that this laboratory provided.

The Regional Surface Laboratory became far more than a shared facility. It embodied Edward’s conviction that advances in materials science depend upon advances in chemical understanding. Throughout his career, sophisticated instrumentation was never an end in itself. Its value lay in the questions it enabled researchers to answer. That philosophy would define the scientific contributions that followed.

## 5. Understanding Interfacial Chemistry Through XPS

X-ray photoelectron spectroscopy became one of the principal tools through which Edward Sacher investigated surface and interfacial chemistry. The ability of photoelectron spectroscopy to provide information about chemical state and bonding had been established during the development of the technique in the 1960s, notably through the work of Kai Siegbahn and his colleagues [12], and its application to surfaces and interfaces subsequently developed through the contributions of many researchers [13]. Edward’s contribution lay in applying and extending these capabilities to chemically and technologically important materials systems. In his laboratory, XPS was used not simply to determine surface composition, but to investigate how atoms were bonded, how surfaces interacted, how interfaces evolved, and how these interactions influenced material properties.

This philosophy shaped virtually every aspect of his research. Rather than asking what elements were present on a surface, he asked how those atoms were bonded, how they responded to changes in processing conditions, and how subtle chemical differences influenced adhesion, stability, electronic behaviour, and reactivity. Throughout his career, XPS became not simply a characterization tool but a means of testing chemical hypotheses.

One of the clearest examples of this approach was his work on the interpretation of asymmetric XPS line shapes in first-row transition metals. Conventional analyses generally attributed these asymmetric peaks to many-body electronic effects described by the Doniach–Šunjić formalism. Edward and his collaborators demonstrated that many of these spectra could instead be resolved into chemically meaningful symmetric components corresponding to distinct atomic environments within the material. These components provided quantitative information about particle size, oxidation, substrate interactions, alloy formation, and surface structure that would otherwise remain obscured. Rather than treating asymmetry as an unavoidable complication, he transformed it into chemically useful information [4].

This conceptual advance did not emerge in isolation. It evolved through a series of carefully designed studies examining transition metal nanoparticles deposited on well-defined substrates. Investigations of transition-metal nanoparticles, including nickel and platinum, demonstrated how subtle changes in XPS spectra reflected differences in particle morphology, chemical environment, and interaction with the underlying surface. Together these studies established a coherent framework for interpreting complex spectra and provided researchers with new ways of extracting chemically meaningful information from XPS measurements [4,5,6].

Although this work required considerable technical expertise, its significance extended well beyond spectroscopy itself. By linking spectral features directly to atomic structure and interfacial bonding, Edward used surface analysis to address fundamental questions concerning materials chemistry rather than merely describe surface composition. This shift in perspective influenced much of his later research and remains evident in many studies that continue to use XPS as a mechanistic rather than descriptive technique.

## 6. Interfaces in the Nanotechnology Era

The emergence of nanotechnology during the late 1990s and early 2000s provided a natural extension of Edward’s long-standing interest in surface chemistry. Nanoparticles possess exceptionally high surface-to-volume ratios, making their behaviour inherently dependent upon interfacial chemistry. Rather than representing a departure from his earlier work, nanomaterials provided an ideal opportunity to extend principles that had already guided decades of research on polymers and thin films [3].

A particularly productive period of his career involved studies of metal nanoparticles deposited on highly oriented pyrolytic graphite, graphene, and carbon nanotubes. These investigations addressed a deceptively simple but technologically important question: how can nanoparticles be attached strongly to carbon materials without compromising their desirable structural and electronic properties?

Edward’s laboratory approached this problem by combining carefully controlled surface modification with detailed chemical characterization. Mild oxidation, plasma treatment, ion-beam modification, and non-covalent functionalization were used to introduce specific chemical functionalities onto carbon surfaces. XPS, Raman spectroscopy, and photoacoustic Fourier transform infrared spectroscopy then revealed how these modifications influenced nanoparticle adhesion and electronic interactions. Rather than relying upon empirical optimization, the work established clear chemical relationships between surface functionality and nanoparticle binding [7,8,9].

Among the most influential discoveries was the demonstration that platinum nanoparticles could be stabilized on graphene through non-invasive functionalization, preserving the desirable properties of the carbon support while promoting strong interfacial interactions. This work illustrated an important theme that recurred throughout Edward’s career: improving materials by understanding the chemistry occurring at their interfaces rather than by modifying their bulk properties [9].

Many of these studies were conducted in collaboration with De-Quan Yang, whose scientific partnership with Edward produced an exceptional series of publications over more than a decade. Together they explored nanoparticle growth, graphene functionalization, carbon nanotube chemistry, nanostructured catalysts, and advanced XPS methodologies. Their work exemplified Edward’s collaborative style. Rather than establishing isolated projects, he cultivated long-term scientific relationships in which increasingly sophisticated questions could be pursued over many years.

The broader significance of these investigations extended well beyond nanotechnology. By combining rigorous surface chemistry with modern nanomaterials, Edward’s laboratory demonstrated that rational control of interfaces could replace empirical approaches to materials design. This philosophy has become increasingly important as materials science has moved toward atomically engineered interfaces and nanoscale functional systems.

## 7. From Synthetic Materials to Biological Interfaces

During the later stages of his career, Edward increasingly applied concepts developed within surface chemistry to biologically relevant materials. This was less a change in research direction than an extension of the same scientific approach into a new class of interfaces.

The connection between these fields was quite direct. Whether the application involved metallized polymers in microelectronics or surfaces designed to interact with biological systems, performance depended critically on the chemistry occurring where dissimilar materials met. In his later work, those interfaces included biological components, particularly in studies related to pathogen detection, wound infection, and antimicrobial surfaces. Questions of adhesion, adsorption, surface modification, and chemical functionality remained central. The interface had changed; the chemistry had not.

Edward’s studies of biomaterials therefore focused on understanding these interactions using the same rigorous analytical methods that had proved successful in earlier work. His later studies increasingly addressed biological interfaces, examining problems such as the immobilization of bacteriophages on engineered surfaces and the formation of protein coronas on nanoparticles. Although motivated by biomedical applications, these studies remained firmly rooted in the principles that had guided his earlier research: understanding how molecular interactions at surfaces govern the behaviour of complex materials [10,11].

This progression reflected one of the defining characteristics of Edward’s scientific career. He rarely abandoned a research question. Instead, he sought increasingly important systems in which the same fundamental chemistry could be explored.

## 8. Building a Scientific Community

Edward’s influence cannot be measured solely through his own publications. Equally important was the scientific community that developed around his laboratory.

Over the course of his career, he authored close to 400 peer-reviewed publications while establishing collaborations that spanned Canada, Europe, Israel, and the United States. Many of these partnerships continued for years, reflecting relationships built upon shared scientific interests rather than individual projects. The Regional Surface Laboratory became an intellectual centre where visiting scientists, graduate students, and collaborators exchanged ideas while making use of advanced analytical capabilities that were uncommon in Canadian universities at the time.

His contributions also extended to scientific leadership. Through the organization of two American Chemical Society symposia devoted to interfacial reactions, Edward helped bring together researchers working across chemistry, physics, and materials science at a time when surface chemistry was rapidly expanding into new technological applications. The resulting proceedings volumes documented the evolution of a field that increasingly recognized the importance of interfaces in determining material behaviour.

Students who trained in his laboratory worked on a breadth of problems. Although individual projects varied widely—from polymers and thin films to nanoparticles and biomaterials—they shared a common expectation that experimental observations should ultimately lead to chemical understanding. Data alone were never sufficient. The objective was to explain why a material behaved as it did and how that behaviour could be altered through control of interfacial chemistry.

## 9. Legacy

Edward Sacher’s scientific career paralleled the remarkable transformation of surface science over the latter half of the twentieth century. During those decades, techniques such as X-ray photoelectron spectroscopy evolved from specialized analytical methods into indispensable tools of modern materials research. Edward not only witnessed that transformation; he contributed to it by applying these techniques to fundamental questions in surface and interfacial chemistry.

His work advanced understanding across an extraordinary range of materials, including polymers, thin films, nanoparticles, graphene, carbon nanotubes, and biomaterials. Yet these diverse systems were united by a remarkably consistent scientific philosophy. Edward approached each new problem not by asking what material should be studied next, but by asking what chemistry governed the interface. It was this perspective—rooted in physical chemistry yet directed toward technologically important materials—that gave his research both coherence and lasting influence.

The laboratory he established, the students and collaborators he mentored, and the body of work he produced continue to influence contemporary materials research. More broadly, his career illustrates the enduring value of approaching complex materials problems through fundamental chemistry. As interfaces become increasingly central to fields ranging from catalysis and energy storage to nanotechnology and biomedical engineering, the questions that motivated Edward’s research remain as relevant today as when he began asking them more than half a century ago.

## Figures and Tables

**Table 1:** Selected stages in the evolution of Edward Sacher’s scientific program.

Period	Major Research Theme	Selected Contributions and Developments	Representative References
1960s–early 1980s	Physical chemistry and polymer materials	Early work in physical and organic chemistry; studies of polymer transport, adsorption, and materials properties during his industrial research career	[1,2]
1980s–1990s	Surface and interface chemistry	Establishment of the Regional Surface Laboratory; polymer metallization; metal–polymer interfaces and applications to microelectronics	[3]
1990s–2000s	XPS and interfacial analysis	Application of XPS to chemical bonding and interactions at surfaces and interfaces; approaches for interpreting complex transition-metal spectra	[4,5,6]
2000s–2010s	Nanomaterials and carbon materials	Metal nanoparticles; chemically modified graphite; carbon nanotubes; graphene; nanoparticle–substrate interactions	[7,8,9]
Later career	Biological interfaces	Extension of surface and interface chemistry to biologically relevant materials, including pathogen detection and nanoparticle–biomolecule interactions	[10,11]

## Data Availability

No new data were created or analyzed in this study. Data sharing is not applicable to this article.
